# Replacing and Healing of Pieces Separated from the Human Body

**Published:** 1882-10-20

**Authors:** G. Halstead Boyland

**Affiliations:** Baltimore, Md.


### REPLACING AND HEALING OF PIECES SEPARATED FROM THE HUMAN BODY.

BY G. HALSTEAD BOYLAND, M.D., M.A., OF BALTIMORE, MD.

The experiment of replacing in position portions of the human body hacked from it is of comparatively recent date. The results have been so far satisfactory as to demonstrate conclusively that such parts, when replaced, do heal, and not only heal rapidly, but bind themselves to the main body with surprising strength and compactness, provided always that two cardinal points be strictly observed: 1st, the piece separated must be kept warm to the normal temperature of the body; 2d, it must be replaced, whether with adhesive plaster or the suture, or both, directly the flow of blood ceases. The following cases are such as frequently come under the observation of medical men abroad.

In the first case, in a duel with schlagers (a weapon something like a rapier, but with a flatter blade, of about the same length and blunt at the end), the left ala with a part of the point of the nose of one of the principals, by a sweep of his antagonist's sword—this piece containing skin, muscles, cartilage and mucous membrane—was cut by a clean wound, square off. It was at once put back into position, sewed on with fine sutures; over the sutures strips of adhesive plaster were applied, extending over the whole point and side of the nose on to the cheeks; in order to prevent evaporation and drying as much as possible, a patch of oiled silk was used, upon which cotton batting was placed, the nose being tamponed also with it at the same time. On the third day the sutures were taken out and the piece found to be quite black; the whole epidermis sloughed off as a black crust, but under it the normal rete malphigii appeared, and one small portion of the epidermis remained. After a time a layer of horny epithelium put out. At the expiration of nine months the wounded man appears with the left nasal ala slightly flattened and of normal color, the surface of the portion that had been cut off made one with the whole side of the nose, no distinct line marking a cicatrix: on some parts of it the epithelium was a little thicker than on others, making a few very small rough places. It is worthy of attention that on the third day when the sutures were removed and the epidermis had sloughed off, the part was firm in its natural position. The sloughing of the epidermis is easily accounted for by the fact that the capillaries became contracted and, so to speak, dead, on account of their extreme fineness, during even the very short time that the piece was separated from the body. We would recommend in such an emergency a process carried out in another case that came under our own observation, viz.: that the separated portion be held in the mouth, if warm water cannot be procured, until the suture and all is ready; thereby the animal heat would be retained and the chances of sloughing of the epidermis materially diminished.

The next case was a student who had fought a duel, also with schlagers, out of whose nose a polygon-like piece was hacked just above and including the tip of the nose, thereby exposing in extenso both nares. This piece was only found after long search, having been thrown by the force of the blow some distance; after the bleeding had ceased it was placed in position. Likewise in this case the greater portion of the epidermis came off in the shape of a black crust. The piece, when healed on, was bordered on all sides by a sharp-edged scar, its color being red and the part itself slightly tumefied. The wound left on the nose at the time was clean and even bordered, as regards the skin, while the cartilage and mucous membrane were separated, irregular and zigzag. The same treatment with reference to detail of placing in position, sutures, adhesive plaster, etc., was carried out in these cases, although the large scar and red color, accompanied by tumefaction, would indicate a less successful result than in the first case mentioned. The length of time that elapsed before the separated piece could be found comes also into consideration, although adhesion takes place more readily after the bleeding ceases; nevertheless, if the parts are left disunited too long, the inclination to adhesion is lost entirely, as it begins to diminish as soon as the main wound commences to dry; the surfaces of wounds of medium size of this character being for some little time moist with a gelatinous substance composed of blood and serum.

As regards the healing itself it is a prima intentio, although the sutures in these cases were only removed on the third day. In treating wounds per primam, in which pieces are not separated from the human body, Bruns removes them in twenty-four hours.

As for the pathological anatomy, or, more properly speaking, the process of healing itself, such cases undoubtedly illustrate that the vessels of the piece, after being placed in position, received in the lumen of each the blood from the severed vessels of the borders of the wound on the nose. In unfavorable cases, a hemorrhagic infiltration of the separated piece, after it is placed in position, which may be followed by mummification, is liable to result. In the more successful ones the epithelial covering is everywhere thrown off. In those again where the surgeon is especially fortunate the circulation in the whole of the portion separated, or in parts of the same, may re-establish itself without any disturbance as to nutrition. Of importance in the healing of wounds upon which transplantation of skin grafting may be carried out, is the proof here deduced, that in transplantation there is a direct flow of blood out of the granulation vessels of the main wound into those of the transplanted piece. In this operation, which is known as Reverdin's transplantation, sloughing of the epidermis is a general rule, which, nevertheless, like all others, has its exceptions; but they are very few and very far between. Technically, where portions of the flesh are severed from the human body, the above procedure is the best to follow; practically, it is the most successful.

In a recent number of the Boston Medical and Surgical Journal is recorded a case in which the hand, almost entirely severed at the wrist, hung to the forearm by a thread of skin only. Instead of amputation the hand was replaced on the above principles and kept firmly in position for a long time; finally it reunited completely, and the patient had considerable use of it, being able to move the fingers. As long as the merest thread connects the divided part to the main limb, so long the circulation may go on in a part of it, gradually re-establish itself throughout and thus save the limb or member, and often the life of the patient.—*Med. Gaz.*

